# Analyzing the politico-moral foundations of the Iran’s health system based on theories of justice

**Published:** 2017-04-08

**Authors:** Forouzan Akrami, Mahmoud Abbasi, Abbas Karimi, Akbar Shahrivari, Reza Majdzadeh, Alireza Zali

**Affiliations:** 1 PhD by Research Candidate, Medical Ethics and Law Research Center, Shahid Beheshti University of Medical Sciences, Tehran, Iran.; 2 Associate Professor, Medical Ethics and Law Research Center, Shahid Beheshti University of Medical Sciences, Tehran, Iran.; 3 Professor, Faculty of Law and Political Science, University of Tehran, Tehran, Iran.; 4 Pharm D, Medical Ethics and Law Research Center, Shahid Beheshti University of Medical Sciences, Tehran, Iran.; 5 Professor, Community Based Participatory Research Center and Knowledge Utilization Research Center, Tehran University of Medical Sciences, Tehran, Iran.; 6 Professor, Functional Neurosurgery Research Center, Shohada Tajrish Neurosurgical Center of Excellence, Shahid Beheshti University of Medical Sciences, Tehran, Iran.

**Keywords:** Politics, Moral philosophy, Public health, Justice, Iran

## Abstract

Public health ethics is a field that covers both factual and ethical issues in health policy and science, and has positive obligations to improve the well-being of populations and reduce social inequalities. It is obvious that various philosophies and moral theories can differently shape the framework of public health ethics. For this reason, the present study reviewed theories of justice in order to analyze and criticize Iran’s general health policies document, served in 14 Articles in 2014. Furthermore, it explored egalitarianism as the dominant theory in the political philosophy of the country’s health care system. According to recent theories of justice, however, health policies must address well-being and its basic dimensions such as health, reasoning, autonomy, and the role of the involved agencies and social institutions in order to achieve social justice beyond distributive justice. Moreover, policy-making in the field of health and biomedical sciences based on Islamic culture necessitates a theory of social justice in the light of theological ethics. Educating people about their rights and duties, increasing their knowledge on individual agency, autonomy, and the role of the government, and empowering them will help achieve social justice. It is recommended to design and implement a strategic plan following each of these policies, based on the above-mentioned values and in collaboration with other sectors, to clarify the procedures in every case.

## Introduction

Public health ethics (PHE) is a relatively new field of applied ethics, and is linked to the ethical implications of activities aimed at maintaining and improving health among the population. PHE covers both factual and ethical issues in health policy and health sciences (1). Article 25 of the Universal Declaration of Human Rights explicitly recognizes the right to health. Article 12 of the International Covenant on Civil and Political Rights emphasizes that governments should recognize everyone’s right to the highest degree of physical and mental health that is possible within each society. Therefore, protecting public health (PH) should be the most important goal of governments. In most countries, the health care system is organized at the national level, which indicates the responsibility of the state. However, these systems suffer from various pathologies that affect their performance (2 , 3). 

PH has affirmative obligations to improve public well-being and reduce evident social inequalities. Therefore, a PHE framework should not only protect the citizens’ negative rights for not intervening, but also emphasize their positive rights (4, 5). Several functional frameworks have been provided by PH professionals to facilitate policy- and decision-making (6 - 8). Any ethical framework has a background of moral theories or at least an ethical approach to justify the selected moral norms, and various moral philosophies and theories can differently shape these structures. For example in consequentialist theories, the policy or action that delivers the best outcome is considered morally right. In utilitarianism, which is one of the most popular frameworks and is widely used in health policy, the only value is to do the greatest good for the greatest number. On the contrary, in deontological theories, the agent’s acts must only comply with moral duties. 

PHE is connected to overlapping spheres of political, social and moral philosophy. However, the health care system mainly requires a “public philosophy”, which would provide a moral foundation and set limits on PH laws, policies and practices, as well as on social institutions and organizations involved in PH activities (9). 

The primary moral justification in PH as an institution is social justice, and the focal points of moral necessities are the oppressed and subordinate groups. These include people whose well-being expectations including health are so limited that their life choices differ from those of others; or children whose prospects of welfare are so poor that they are permanently locked in the systematic deprivations of their early years (10). Will Kymilicka states:

*“Political philosophy is a matter of moral argument, and moral argument is a matter of appeal to our considered convictions. In saying this, I am drawing on what I take to be the everyday view of moral and political argument, that is, we all have moral beliefs; these beliefs can be right or wrong, we have reasons for thinking they are either right or wrong, and these reasons and beliefs can be organized into systematic moral principles and theories of justice. A central aim of political philosophy, therefore, is to evaluate competing theories of justice to assess the strength and coherence of their arguments for the rightness of their views” (* 11 *).*

Rajabi et al. in their study aimed to explain the principles and values of the health system to be utilized in Iran’s health system reform plan of 2025. While emphasizing respect for human dignity and protection of human prosperity, they concluded that addressing PHE challenges necessitates new perspectives on both individuals and the society and the relationship between them (12). 

Designing an ethical framework for health policy-making first requires an analysis of the political philosophy of the country's health care system, since the approach to PH depends on the political philosophy of each country (13). Therefore, this study aimed to explain the political philosophy of the health care system based on theories of justice. In this study, after an overview of these theories, we analyzed the general health policies (GHPs), which have been codified by the leader of the Islamic Republic of Iran in implementing Paragraph I of Article 110 of the constitution after consultation with the Expediency Council.

## Method

Document analysis is a systematic approach for reviewing or evaluating texts. Like other analytical methods in qualitative research, document analysis requires that data be examined and interpreted in order to extract meaning and insight, and develop empirical knowledge (14). In this document analysis, The GHPs document was examined to explain the politico-moral foundations of the health care system based on theories of justice and to explore how the moral values were enfolded. Additionally, for critical discussion, Web of Science (ISI), PubMed, Embase, and Scopus databases were purposefully searched using the following keywords: “public health/ethics” [MeSH Terms] OR “public policy/ethics” [MeSH Terms] NOT “research” [MeSH Terms] AND “philosophy/ethics” [MeSH Terms].

**Theories of** **justice**

*The* *Libertarian Justice Theory*

Influenced by philosophers like John Locke and Robert Nozick, a libertarian theory of justice focuses on individual freedom, and thus on our duty to respect the freedom of others, and the duty of governments to protect the freedom of citizens – as their right – when they are at risk. This often means a “minimal state” to prevent or punish breaches of personal boundaries, including individual property rights. In this view, health care is not a right, but people can voluntarily choose the charitable act and contribute in some way to distribute health care in a community. PH can be legitimate, especially if it focuses on protecting people against infectious diseases; a type of boundary violation, preferably to broader concepts of health promotion that is characteristic of contemporary public health (15). 

Libertarians do not oppose to utilitarian and egalitarian distributive patterns, provided that they are chosen freely. Any fair distribution can be justified in health care coverage, if and only if people have freely chosen it in the relevant groups. As a result, libertarians generally support those health care systems in which health care insurance is private and purchased voluntarily. In such systems, the investors have the property right in the health care of the insured, doctors have the freedom right and the society is not morally obligated to provide health care. The libertarian interpretation of justice is not based on addressing the citizens’ health needs or the general benefit, but rather on carrying out unrestricted fair activities (16). 

*The* *Utilitarian Justice Theory*

Utilitarian theories of Justice were formed by prominent figures such as Jeremy Bentham and John Stuart Mill. The ground conceptions of justice in the principle of utility require policies, actions or rules that produce the maximum benefit. Justice, which involves the correlation of rights and duties, is not an independent decision, but rather a derivation of interest. Within this framework, the duties and rights in fair health care are the presupposition of net profit foundation. Health care and PH can be valuable at least to the extent that they produce net social benefit (15). Most of the utilitarian social programs support PH and distribute basic health care among all citizens. Nevertheless, rights such as health care – when based on maximizing the ultimate good – will find a fragile basis, because the benefits may change at any time. For example, it seems unfair that a society itself maximizes the ultimate good by eliminating the access of the weakest and the sickest population. Therefore, utilitarian principles of justice seem to have very serious problems, but if their inclusion scope is strictly limited, they can play a major role in health policy-making (16). 

*The* *Communitarian Justice Theory*

The communitarian justice theory arises from several philosophical views similar to utilitarian theories and do not assign an independent importance to individual rights such as freedom. Thus, the perception of health care and a just health system depends on the community perception of health in relation to other primary goods (15). Communitarians have a pluralistic view on the principle of justice, believing that they are as varied as the diverse perceptions of good in different societies. The duty of people with respect to justice depends on the criteria in each community. Communitarians emphasize both the duties of the society towards individuals and the duties of the individuals towards the community. Some communitarians avoid using the language of justice and use one of unity and integrity that includes both the values related to individual obligations and the principles of social ethics based on the common beliefs of a group. Justice concepts do not rise from the rational or natural principles outside the community, but from criteria that are shaped internally along with the political development of the society. Communitarians believe that emphasis on the community and the common good in health care allocation policies is also evident (16). In this regard, Daniel Callahan says that “we need to ask what can best guide us towards a good society, rather than whether it is harmful or whether it violates the autonomy of the people” (17). 

*The* *Egalitarian Justice Theory*

Egalitarian theories draw on old religious perspectives that believe all human beings should be treated as equals in certain respects because they are created equal (16), and this makes the foundations of human rights (18). No prevailing egalitarian theory has been exclusive of a distributive principle based on equal sharing of all primary goods by everyone. It is characteristic of the dominant egalitarian theories to identify basic equalities that allow for some inequalities (16), and many of them recognize the possible legitimacy of a two-layered system, with the minimum decent layer of health care (set by the deliberative democracy).

John Rawls’ Theory of Justice is the mildest, most important egalitarian theory that has challenged liberalism, utilitarianism and communitarianism. Among those who have been influenced by John Rawls, Norman Daniels argues that justice requires the elimination or reduction of obstacles that prevent fair equality of individuals’ opportunities, including health as a moral importance, to allow people to pursue a variety of objectives and programs of life depending on their talents and skills. This includes programs to compensate for the shortcomings of people such as health deprivation. Daniels looks for a comprehensive plan for fair health care and investigates the role of social determinants of health such as education, environmental and behavioral factors, and the socioeconomic status of communities (16, 19). 

With the start of the 21^st^ century, some innovative ideas raised debates about justice in the field of biomedical ethics. Although this article has been formed in response to Rawls’s egalitarian theory, it is not entirely the same in fundamental terms. It is mainly influenced by the ethical theory of Aristotle, especially the role and importance of human flourishing states that rely mostly on fulfillment and moral virtue. In the following section, some recent theories of justice will be discussed. 

*The* *Capabilities Theory*

This theory is based on the assumption that the opportunity to reach states of proper functioning and well-being is an object of value and moral importance, and thus capability reflects an individual’s autonomy in selecting one of several alternative lives. People’s quality of life is conditioned by what they are able to do, and a life well lived is one in which people perform and maintain their basic capabilities. This theory was first proposed by Amartya Sen, and developed in many ways related to biomedical ethics by Martha Nussbaum. The latter explored the philosophical concept of “frontiers of justice” to address equitable inclusion for persons with disabilities, the poor, and animals. The central idea is that the minimum level of social justice entails the availability of 10 core capabilities for all citizens (16), which means everyone should be able to: 

1) lead a normal life without encountering premature death or a deteriorated state unworthy of living

2) have the benefit of physical health

3) enjoy bodily integrity, that is, the ability to live in freedom and have security against violence, sexual satisfaction and fertility choice

4) use the capacity of senses, imagination and thought

5) enjoy emotional attachment to people and other entities and experience a feeling of gratitude

6) apply practical reasoning and participate in serious reflections bearing on one’s life arrangements

7) feel organizational affiliation as the capability to lead a meaningful life in cooperation with a company or others

8) be free to exhibit concern or care for other species

9) play and enjoy creative activities

10) have control over one’s environment as an active citizen

*The Well-Being Theory*

The capabilities theory focuses on abilities and opportunities as prerequisites for well-being, but more recent theories have focused on well-being itself. In other words, freedom of action, capabilities, the associated empowerment trainings and resources are considered the well-being equipment (16). Powers and Faden have formulated a framework for bioethics in PH and health policy by providing a non-distributive theory of justice that complements distributive justice and goes beyond it. They believe that questions about important inequalities can only be answered by examining all the social determinants that increasingly and mutually impact human well-being. According to Powers and Faden, justice is more than the principles of distribution (10, 16) and beyond the distributive share of each person, and is identically connected to the nature of the relationships between individuals. Some topics of discussion in the area of justice both for individuals and for groups include: concerns about social stigma, disrespect, lack of organization and social functions for adequate protection of existing capacities to maintain social independence or autonomy (20). From this perspective, the aim of justice is to ensure an acceptable level of the six basic dimensions of well-being, including health, reasoning, self-determination, attachment, personal security and respect for all (16, 21). 

Citizens of countries that lack a comprehensive and coherent system of health care finance and delivery are unfortunately deprived of health services in spite of spending high costs. It is the obligation of governments to promote both utility and justice in the society (16). For this reason, we will analyze the general health policies (GHPs) of Iran served at the national level based on the aforementioned theories. 

**Analysis and criticism of general health policies** **in Iran**

As the first item of the Iranian GHP, “beneficence and service delivery based on Islamic human values, spirituality and moral virtues ​​and their promotion in the community” have been emphasized. These issues are clearly linked to social justice, and whenever people are in the position to do good things or impose costs, they will need justice criteria (16). In the context of public health, justice is a core ethical consideration, but unfortunately a theory of Islamic justice is presently lacking. 

In recent decades, significant advances have been made in the field of primary health care, academic education and research in Iran. Progress in biomedical research has been accompanied by significant activities in legislation, education and research in the field of bioethics (22). However, the first paragraph of the first policy highlights “evolution” in academic environments in accordance with Islamic values, medical ethics and professional practice. This implies the poor desirability of the current situation in service provision and the need to reform the health care system based on Islamic moral values. ​​Moreover, the second paragraph of this policy emphasizes the importance of educating people about their rights and social responsibilities, and utilization of the full capacity of health care environments for the promotion of Islamic ethics and spirituality in the society. 

The second, fifth, and sixth policies of the GHPs directly point to egalitarianism, equitable access and fair distribution of health care services based on people’s needs. These sections highlight access to health as a social right, the responsibility of the state to make it happen, and egalitarianism as the dominant theory in the political philosophy of the national health care system. For the purposes of accountability, realization of justice, and provision of desirable medical services, the seventh and eighth policies specify that health resources be managed through the health insurance system, and that services be delivered by both public and private sector service providers in accordance with the legal provisions. The eighth policy further emphasizes the significance of the principle of justice and the importance of accountability, transparent informing, effectiveness, efficiency and productivity in the health care system in keeping with evidence-based ranking and the referral system. The third paragraph of the eighth policy concerns the protection and care of veterans and the disabled community as vulnerable members of the society, and concentrates on empowerment and the promotion of health among them. 

The third policy focuses on the importance of a healthy lifestyle, and the twelfth policy pertains to healthy nutrition based on traditional Iranian medicine, while the fourth policy is dedicated exclusively to the quality and efficiency of services. The eleventh policy is related to “raising awareness, responsibility, empowerment, and active and structured participation of the individual, family and community to provide, maintain and improve health by using the capacity of institutions and cultural, educational and media organizations under the supervision of the Ministry of Health and Medical Education”. It is obvious that this is a move aimed at achieving social justice, and therefore cannot be placed under the supervision of the Ministry of Health and Medical education alone. 

The ninth policy discusses measures including: fair distribution through qualitative and quantitative development of health insurance and its delivery to the public; complete coverage of the basic treatment needs of the people by providing insurance to the whole of the society and reducing the share of the insured in medical costs; and providing services beyond basic insurance through complementary private coverage within the framework of legal and transparent stipulations with an emphasis on high quality basic health services. Moreover, paragraph 7 of the ninth policy proposes a number of supplemental mechanisms to ensure public health, for instance reforming the performance-based payment system, raising efficiency, creating fair income and positive motives for service providers, and special attention to health promotion and preventive activities in deprived areas. One problem associated with health related goods and services concerns determination of the precise limits of the right to health. An approach in this respect is equal access to health resources. From a minimalistic standpoint, this means public access to health care, which is in accordance with the idea of some libertarians regarding the right to public resources, although this view is untenable by the justice theories previously mentioned. For this reason, current prominent liberal societies have created significant progress in solving the challenges facing their health care system in connection with the access, affordability and quality of care. As an instance, the United States approved the Act of Affordable Care (ACA), and since its adoption, the total rate of the uninsured has decreased by 43%, from 16% in 2010 to 9.1% in 2015 (23). Therefore, the aim of moderate egalitarianism may be defined as the right to minimum, decent health care, that is, public access to basic health care and related resources. The standard concept, however, requires a two-layered system as follows (16): 

1. Compulsory social coverage for basic health needs and common mishaps 

2. Voluntary private coverage for other health needs and demands 

The first layer addresses health needs through public access to basic services. This model of a pure protection to all indicates that social requirements can be limited, which necessitates the definition of basic and secondary health needs based on the social norms in each society (19). 

The tenth policy pertains to “sustainable financing in the health sector” and indicates the prioritization of public health by the state. The third paragraph of this policy discusses “imposing taxes on products and materials, as well as punishments on harmful health services”, which refers to the important role of PH law in its implementation and continuation. Moreover, the fourth paragraph covers “paying subsidies to the health sector, targeting health subsidies and treatments aiming at justice and the promotion of health particularly in deprived areas, and providing dedicated help to poor people and lower income groups”. This latter point clearly emphasizes the fairness of needs-based distribution and fair financial contribution. 

The thirteenth and fourteenth policies imply the importance of the educational aspect of medical sciences in providing efficient local and national human resources in order to improve the overall health of the population (24). These policies also stress the strategic development of medical research with an innovation and planning system approach to achieve excellence in science, technology, and provision of medical services in the region and throughout the Islamic world, in accordance with the country’s 20-year vision document and to complement the previous policies. 

## Discussion

To a large extent, the principles of egalitarian justice, respect for everyone and treating all people as equals comply with the fair procedures doctrine in the distribution of primary goods and not only health care. In the second policy, reference to “*realization of a comprehensive approach to health and a healthy society in all legislation and executive policies*” is consistent with the approach to health in all polices. The implementation of this approach enables the state to establish an integrated act in response to the health needs and well-being of the people. This, together with the ultimate goal of reducing health inequalities, considers the effects of other policies and laws on health through the social determinants of health (25), and will be realized when health-based laws and policies are designed, planned and implemented in all areas (26). 

No dominant egalitarian theory that consists of a distributive principle has been based on equal sharing of all primary goods by everyone. According to Daniels, allocation of health care resources should provide justice through fair equality of opportunities. This inspiring theory of Rawls has broad implications in national policies of health care. Based on this theory, each member of the society should have enough access – not necessarily maximum access – to a level of health care regardless of their assets and positions. The exact level of access depends on the available social resources and public processes for decision-making. Daniels believes that the social institutions influencing the distribution of health care should be coordinated so as to allow each person to receive a fair share of the normal range of the opportunities available in the community to pursue the objectives and plans of his or her life (16, 19). 

According to the theories of “Moral Desert”, people get what they deserve: good people are rewarded and bad people are penalized (27). From the perspective of defenders of luck egalitarianism, paying higher premiums or taxes by people who have healthier choices is not fair when others may have had morally irresponsible behaviors (28, 29). They believe that discussions on social factors affecting health are untenable. Such reasoning is applied to health services in relation to crimes or bans on the access of individuals or groups who choose unhealthy lifestyles. Nevertheless, there are reasons for unhealthy behavior that are not mere choices (30). Differences in choices and lifestyles can be caused by social conditions and inequalities (31). Some characteristics are the consequence of the natural and social lottery, and many people do not have a fair chance to obtain or change them; hence, they cannot be a morally acceptable basis for discrimination in allocation of social resources. Accordingly, people with disabilities should receive a higher level of health care to have a fair chance in life. They may not be entitled to health care services if they are responsible for​​ their disability, but if they are not, the principle of fair opportunity requires that they receive services that help them to compensate for the negative effects of lotteries (16). 

In proposing such an approach, Sen has presented an explicit critique of Rawls’ “fair equality of opportunity”, arguing that such opportunities are brutal stories, since many options are the outcome of poverty, low literacy, racism, and other similar events that are determined at birth. He argues that the primary and main concern of justice must be something that people are really able to achieve, a situation that Amartya Sen calls “substantive freedom”. In the view of Sen, this includes “the ability of a person to do good actions and reach valuable states of being” (32). If people’s abilities are restricted by the circumstances or a range of limited conditions, the society cannot be considered a just one (33). In this view, injustice can be evaluated by the current practices and policies of major social institutions within the community. Such practices enable certain social groups to develop capabilities necessary to obtain a decent and reasonable life, find work or other living arrangements to support their family, and be employed in projects, activities and valuable social relationships. 

There are occasional conflicts between public interests and autonomy in policy debates of public health that can be resolved by concentrating on the difference between freedom and autonomy. The debates on the potential violation of the principle of autonomy need to refocus on the issue of whether people can make meaningful choices about what they can do in their lives. People may be free to buy a large bottle of carbonated drink, but we cannot be dragged into talking about this deviated way of respecting autonomy, nor should we think that making the purchase of extra-large harmful products difficult is an important step in improving the lives of people and creating a just society (34). 

Although there is not a single theory of justice that is unanimously accepted, “social justice” is a common term in the field of public health these days (35). The recent theories of justice state that having freedom to choose healthy behavior is not enough, and the ability of individuals to reason and their autonomy to make healthy choices must be developed (16, 21). David R. Buchanan argues that improving public health is better achievable by expanding people’s autonomy through promoting the concept of justice, which is the definition of human progress. According to recent theories of justice, the most important issue in PH is not limiting the autonomy of individuals, for example by restricting the access of minorities and the poor to fast food or sugary, carbonated drinks in the hope of their weight loss, but rather the promotion of autonomy among community members. In other words, autonomy is a core value in a just society in which conditions for fostering the abilities of reasoning and decision-making are provided (34). Schröder-Bäck et al. analyzed the health strategy of European Union (EU) via its ethical scope and considered implications for future health policy-making. Their study showed that the health strategy of European Union is barely documented and discussed in scientific literature, and that no specific attention has been given to its value base. Their analysis showed that the mentioned values are particularly focused on health care in general rather than on PH in particular. They also concluded a theory of well-being is needed on a more general level for effective policy-making. Therefore, a moral theory is required to explain this and the place of health values in a comprehensive and coherent policy approach (36).

Although the capabilities theory highlights the important considerations in the analysis of the relationship between agency and structure, according to the theory of well-being, the aim of justice should be to ensure an adequate level of basic welfare, including health, reasoning and autonomy for every person, and not only the capabilities and means to achieve it. In this doctrine the origin of value or obligation is not merely individual choices or exercising one’s intellectual capacity for choosing, but the process of “creativity through choosing” which is integrated in autonomy is considered a part of well-being. PH professionals need to pay attention to much stronger fields, both moral and practical, in advocating for health policies and programs rather than creating bans (34). 

A healthy lifestyle is influenced by both individual agency and social determinants of health, including structural factors and living conditions (31). Recently, personalism has been proposed firstly in regard to human dignity and secondly because of the agency of individuals as social beings who construct the collective good through solidarity, and as the philosophical core of the health care system (2). The concept of lifestyle-related diseases and individual responsibilities for/toward health plays an important role in discussions about fair allocation of scarce health resources. Looking into this issue from the perspective of solidarity emerged as a value in the context of a Solidarity Project in Bioethics by Nuffield Council in 2011. Barbara Prainsack has analyzed the most important arguments in favor of using lifestyle choices as a benchmark in solidarity-based health policy-making to prioritize and classify access to health care services (30, 37, 38). Still, arguments about crimes or prohibitions on the access of individuals or groups that choose unhealthy lifestyles are provided in treatment services. Nevertheless, there are different unhealthy behaviors that might not be restricted to “mere choice”, so access to PH based on lifestyle choices is not an exclusively moral issue, and despite the arguments presented to this effect, unhealthy behavior is not a breach of solidarity in itself (30). 

Recent studies have explained the role of moral virtues ​​in PH (39 - 41). They demonstrate that for the past several decades, the concept of “structure” in moral theology has almost exclusively focused on the structure of the society with regard to the need to change. The structures that have continued unfair positions and created systematic barriers for human development are classified as “structures of sin” and have therefore been the object of social and theological criticism. PH professionals and health policy-makers are attempting to create new structures (i.e. law, policy and environment) that have a positive impact on the lives of individuals and communities. Such social structures are formed by individual characteristics and virtues as units of fundamental value that form each person’s habits and behaviors. According to Michael D. Rozier, “structure is only one part of a larger system of our behavior” (40). We have the disposition rooted in our personality and we want to cultivate it. We identify the habitual behaviors that are transformative, and adopt social norms that encourage the behavior. Therefore, we build the social structures that promote social norms and virtues. In this manner, the internalized virtues of moral agents continue to spread across the community and ﬁnally to structures that shape the society and agents (40).

The constitution determines the special powers of the federal government and limits its authority to protect freedom (42). In other words, the constitution of a country provides a framework for the localization of global treaties including the International Declaration of Human Rights. Although the state has the authority to act for the common good, it should also apply the internal power of the limits imposed by the constitution (21). The Charter of Fundamental Rights of the European Union has been developed in accordance with the common commitments between international and national laws of the European Union in line with the citizen’s rights of member states, including the right to health care (43). In Iran, the constitution, the 20-year vision document, and the comprehensive scientific map of health are among the reference documents that have presented the guidelines for involved institutions. 

The first step towards awareness of a law that can change people’s lives is legal literacy. Legal literacy programs educate community members, patients and health care providers about their national and local laws and their rights, and this knowledge enables them to utilize these rights and seek support for specific health needs. Some objectives of such programs include: increasing awareness and capacity building, training educators, education and community empowerment, and encouraging law students to work for social justice and solidarity (44). These goals are all considered as moral norms in PH and health policy-making (45).

## Conclusion

Given the centrality of the principle of justice in public health, in this study we analyzed GHPs issued at the national level in terms of the theory of justice. The findings point to egalitarianism as the dominant theory in political philosophy in the country’s health care system. 

The first policy on the list focuses on beneficence and providing health services based on humanistic/Islamic culture and values ​​and their institutionalization in the community without mentioning the fair procedures. Although decision- and policy-making in the field of bioethics has no justification in Muslim societies without paying attention to the Islamic culture, justice principles are required in providing goods. The second, fifth, and sixth policies of the 14-item list of GHPs directly point to egalitarianism, equitable access of people and fair distribution of health care services based on need. Nevertheless, since “social justice” adequately supplies well-being dimensions including health beyond distributive justice, the distinct areas of justice are rejected. It is possible, however, to talk about justice in the PH and health policy-making without referring to the construction of other public policies and social structures. These policies must therefore address something beyond well-being and its basic dimensions such as health, reasoning and autonomy, or the role of agencies and involved social institutions in order to achieve social justice. Making people aware about their rights and responsibilities, as well as increasing their knowledge and empowerment, implies the role of individuals’ agency and autonomy in choosing their lifestyle, in addition to the role of governments in achieving social justice. Finally, for the institutionalization of humanistic/Islamic values ​​in the community, public health structures should aim to promote healthy behaviors. Moreover, for the purpose of policy-making in the field of health and biomedical sciences in Islamic communities based on Islamic culture, a theory of social justice in the light of theological ethics is essential.

Community participation requires transparency, commitment and responsiveness of health care providers, and solidarity is a core value based on moral responsibility and virtues that support and sustain PH policies, programs and interventions. Therefore, it is recommended to design and implement a strategic “how to” plan following each of these policies based on the above-mentioned values and in collaboration with other sectors. 
